# Comparing Cognitive and Physical Limitations as Predictors of Depression Among Older Adults

**DOI:** 10.1093/geroni/igab046.3106

**Published:** 2021-12-17

**Authors:** Clara Scher, Takashi Amano, Lenna Nepomnyaschy

**Affiliations:** 1 Rutgers University, New York, New York, United States; 2 Rutgers University-Newark, Newark, New Jersey, United States; 3 Rutgers University, New Brunswick, New Jersey, United States

## Abstract

Depression in older adults is associated with loss of functioning and increased mortality. While many factors contribute to depression among this population, activities of daily living (ADL) limitations and cognitive impairment have been identified as key risk factors. However, no study, to our knowledge, has examined the extent to which physical and cognitive limitations independently and jointly contribute to the risk of depression. The current study describes the prevalence and compares the independent and joint associations of these limitations with depression in a nationally representative sample of adults aged 51 and older in the US. Analyses are based on a sample of 17,044 repeated observations on 6,636 unique primary respondents from three waves of pooled data from the Health and Retirement Study. We estimate linear and logistic multivariate regression models investigating the association between ADL limitations (any limitation on Katz ADL scale), cognitive impairment (<12 on the TICS-27 scale), and depressive symptoms (8-item CES-D), controlling for a standard set of socioeconomic and health factors. First, we find that 66% of respondents report no limitations, 16% report only cognitive impairment, 11% report only ADL limitations, and 7% report both types of limitations. Multivariate analyses suggest that ADL limitations have a much stronger association with depression compared to cognitive impairment, and this association is robust across alternative specifications. In next steps, we will take advantage of the longitudinal nature of these data to estimate changes in these characteristics over time and within individuals and explore heterogeneity in associations across relevant groups.

